# Making the most out of timeseries symptom data: A machine learning study on symptom predictions of internet-based CBT

**DOI:** 10.1016/j.invent.2024.100773

**Published:** 2024-09-12

**Authors:** Nils Hentati Isacsson, Kirsten Zantvoort, Erik Forsell, Magnus Boman, Viktor Kaldo

**Affiliations:** a Centre for Psychiatry Research, Department of Clinical Neuroscience, Karolinska Institutet, & Stockholm Health Care Services, Region Stockholm, Sweden; b Institute of Information Systems, Leuphana University, Lueneburg, Germany; c Division of Psychiatry, University College London, UK; d Department of Medicine Solna, Clinical Epidemiology Division, Karolinska Institutet, Stockholm, Sweden; e Department of Psychology, Faculty of Health and Life Sciences, Linnaeus University, Växjö, Sweden

**Keywords:** Machine learning, Prediction, Treatment outcome, Adaptive treatment strategy, Precision psychiatry, Timeseries symptom

## Abstract

**Objective:**

Predicting who will not benefit enough from Internet-Based Cognitive Behavioral (ICBT) Therapy early on can assist in better allocation of limited mental health care resources. Repeated measures of symptoms during treatment is the strongest predictor of outcome, and we want to investigate if methods that explicitly account for time-dependency are superior to methods that do not, with data from (a) only two pre-treatment timepoints and (b) the pre-treatment timepoints and three timepoints during initial treatment.

**Methods:**

We use 1) commonly used time-independent methods (i.e., Linear Regression and Random Forest models) and 2) time-dependent methods (i.e., multilevel model regression, mixed-effects random forest, and a Long Short-Term Memory model) to predict symptoms during treatment, including the final outcome. This is done with symptom scores from 6436 ICBT patients from regular care, using robust multiple imputation and nested cross-validation methods.

**Results:**

The models had a 14 %–12 % root mean squared error (RMSE) in predicting the post-treatment outcome, corresponding to a balanced accuracy of 67–74 %. Time-dependent models did not have higher accuracies. Using data for the initial treatment period (b) instead of only from before treatment (a) increased prediction results by 1.3 % percentage points (12 % to 10.7 %) RMSE and 6 % percentage points BACC (69 % to 75 %).

**Conclusion:**

Training prediction models on only symptom scores of the first few weeks is a promising avenue for symptom predictions in treatment, regardless of which model is used. Further research is necessary to better understand the interaction between model complexity, dataset length and width, and the prediction tasks at hand.

## Highlights

• Early prediction of patients at risk of not benefitting enough in ICBT can help adapt treatment and allocate resources
• Including data from the initial treatment period improves predictions compared to pure baseline prediction
• Machine learning models for repeated measurements can be clinically useful but are as accurate as simpler prediction models
• More complex models that account for repeated data might need more data or optimisation to be superiour

## 1 Introduction

Addressing the need for evidence-based psychological treatments, Internet-Based Cognitive Behavioral Therapy (ICBT) is now increasingly used in regular care (Titov et al., 2018). ICBT has been found to have a similar effect as face-to-face CBT and comes with benefits such as independence of time and place (Andersson et al., 2019). Yet, 30–60 % of patients do not benefit sufficiently from treatment (Rozental et al., 2019), causing researchers such as (Karyotaki et al., 2021) to call for more personalized treatment choices. However, how to best address patients' individual needs with the limited resources available remains an open question (Vieira et al., 2022). Predicting treatment outcomes holds promise for individual treatment adaption (Forsell et al., 2019; Hornstein et al., 2023), with initial results showing promising effects from such adaptions (Forsell et al., 2019) similar to, or possibly stronger, than results from similar treatment strategies in traditional psychological treatment, often labeled Routine Outcome Monitoring (Barkham et al., 2023; de Jong et al., 2021). Additionally, the ease of data collection in ICBTs opens the door for powerful advanced analytics, such as Machine Learning (ML) models.

Various data types have been considered for outcome predictions, ranging from neuroimaging, socio-demographic, and genetic to behavioral data (Lee et al., 2018; Vieira et al., 2022; Wallert et al., 2022). Several of these features are expensive and time-consuming to obtain, making them a challenging choice for large-scale routine care settings. To further complicate the matter, collecting data about mental health requires a diligent approach of data minimalism to protect patients' privacy rights (Cote-Allard et al., 2022). Such challenges could be one reason a recent review found that only 3 % of the investigated interventions for depression already use ML for personalization. At the same time, they report that symptom data was the most important variable for human-made stepped-care decisions (Hornstein et al., 2023). These findings concur with (Forsell et al., 2020; Hentati Isacsson et al., in press), who found symptom data to be the most critical predictor in outcome predictions. Gathering symptom measurements every week is common practice in ICBTs (Hornstein et al., 2023; Titov et al., 2018), which makes them readily available at scale. If one wants to predict the final score on a symptom measure, using the early treatment trajectory of that same measure is a promising candidate for outcome prediction (Forsell et al., 2020). Furthermore, symptom data is simple to anonymize and – in contrast to, for example, genetic data – only holds for the moment at hand, limiting their potential malevolent use.

As symptom data is gathered over time, it creates a hierarchical dependency in the data. Nevertheless, most studies so far only use time-independent models such as simple Linear Regression, Support Vector Machines, and Tree-based algorithms (Lee et al., 2018; Sajjadian et al., 2021; Vieira et al., 2022). As these models cannot account for the time aspect, the data are gathered as means, sums, or independent features, introducing a possible bias (Afshartous and de Leeuw, 2005). Therefore, a key aim of the current study is whether accounting for the dependence of repeated measures will improve outcome predictions.

Time-series modeling is a field of study that concerns itself with the very topic of time-dependent data. However, in this field, much longer input sequences are standard than the shorter sequences commonly found in ICBTs, e.g. Titov et al. (2018). For inferential research in psychotherapy, data hierarchies are often handled using multilevel models (Hesser, 2015). In ML, many neural network models can account for time-dependent input, but they require large datasets to perform well (Bzdok and Meyer-Lindenberg, 2018; Ebert et al., 2019). Sajjadian et al. (2021) found that out of the 56 studies looking into outcome prediction for depression, only 25 (44 %) had a greater sample size than 100.

In summary, symptom features offer themselves for outcome prediction as they are the strongest known predictors, efficiently obtained on a large scale, easy to anonymize, and can be used for treatment adaptation (Forsell et al., 2020). However, so far, the models used do not leverage the time-dependency of therapy trajectories. While time-dependent model options are ample, it is unclear whether they will yield a benefit in the context of ICBT predictions, due to their requirements regarding sequence length and dataset size.

### 1.1 Objectives

The aim of this paper is to compare prediction methods that explicitly account for the time-dependent structure of data with methods that do not, when predicting symptom treatment trajectory in ICBT. We will also investigate how different lengths of sequential predictor data impact the model's performance. To this end, a) a shorter training sequence of only two timepoints before treatment start will be compared to b) a treatment period, providing five timepoints for prediction. The dataset consists of high-quality clinical data of 6436 patients in regular care, which is 55 times larger than the median dataset in outcome predictions in mental health (Sajjadian et al., 2021).

## 2 Methods

**Fig. 1-:**
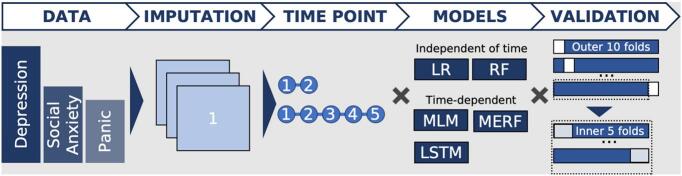
Datasets and analyses Detailing datasets and analyses. After multiple imputation (3 imputation sets) the data is aggregated into one dataset containing all treatments and only primary symptoms from each treatment. For each imputation set, the data is then split based on training sequence length (2 or 5). The analyses for each model are tuned and validated with 10 × 5 folds, where tuning is done on the inner 5 folds. LR, Linear Regression. RF, Random Forest. MLM, Multilevel Model regression. MERF, Mixed Effects Random Forest. LSTM, Long Short-Term Memory: a recurrent neural network architecture.

The study is a prediction study utilizing observational longitudinal data from a regular care clinic to predict individual symptom trajectories and compare prediction models. Ethical approval was received from the regional ethical review board in Stockholm (Dnr: 2011/2091–31/3, amendment 2016/21–32, 2017/2320–32 and 2018/2550–32). The following section details the analysis based on the steps shown in Fig. 1.

### 2.1 Setting, participants, and data

The participants (*n* = 6436) were routine care patients at the Internet psychiatry clinic in Stockholm, Sweden between January 2008 and February 2021 (Titov et al., 2018). They received 12 weeks of ICBT for either major depressive disorder (*n* = 2897), panic disorder (*n* = 1633), or social anxiety disorder (*n* = 1906). The treatments are guided by a licensed clinical psychologist and have shown positive results (El Alaoui et al., 2015; Hedman et al., 2013, Hedman et al., 2014). For more details on the dataset and its context see Hentati Isacsson et al., (in press).

Each treatment consists of condition-specific established CBT techniques, including different types of exercises, and weekly self-assessments of the primary symptoms. The symptom questionnaires, and thus the primary continuous outcome for each treatment, were: Montgomery-Åsberg Depression Rating Scale-Self report (MADRS-S) (Montgomery and Asberg, 1979) for major depressive disorder, Panic Disorder Symptom Scale-Self Report (PDSS-SR) (Houck et al., 2002) for panic disorder, and Leibowitz Social Anxiety Scale-Self Report version (LSAS-SR) (Fresco et al., 2001) for social anxiety disorder. Assessments were conducted at screening, before treatment start, on a weekly basis 10 times during treatment, and after treatment, resulting in 13 timepoints. While the weekly assessments are 1 week apart, there are some variations in when patients complete the assessments, making the timepoints not exactly equally spaced in time. To investigate the predictive power of the time-dependency in the data, a minimal data approach was used which means that each dataset only contains the primary symptom scores as variables. Thus, the only predictive variables used are the symptom questionnaire sum for each week and dummy variables indicating the original treatment condition. This was the same for all models, for more details see the supplement codebook.

Previous research investigated the possibility of pooling the data of different treatments into one dataset and found this to be favourable (Hentati Isacsson et al., in press; Zantvoort et al., 2024). To account for different scales of the symptom questionnaires a min-max transformation (Cohen et al., 1999) based on the questionnaire min and max, was applied to each intervention individually. Min was 0 for all three questionnaires and max was: 28, 54, 144 for PDSS-SR, MADRS-S, LSAS-SR, respectively.

#### 2.1.1 Predicted outcome

The predicted outcome was the continuous summed score of the primary symptom measures as detailed above. To evaluate and compare models using both continuous performance measures as well as binary performance measures, the continuous prediction was dichotomized in retrospect. After the models predicted the continuous outcome, the score was dichotomized into ‘success’ if either of two conditions (R∪S) were fulfilled. Firstly: the score (s) fell below remittance for the scale, $R=s<r_{t}$, where the remitter scores ($r_{t}$) were 11 for MADRS-S (Fantino & Moore, 2009), 8 for PDSS-SR (Furukawa et al., 2009) and 35 for LSAS-SR (Glischinski et al., 2018). Secondly: if the score (s) indicated a sufficient symptom reduction of 50 % (Karin et al., 2018) from pre-treatment ergo,$S=s\leq s_{p}\cdot 0.5$, where $s_{p}$ is the pre-treatment score. Thus, the dichotomization of the score is defined by R∪S and occurred after predicting the continuous outcome, with no separate dichotomization of the outcome itself. See Hentati Isacsson et al., (in press) for a complete description.

#### 2.1.2 Prediction models

The following models were used for analyses all implemented in python v 3.8.10: Linear regression (LR) and random forest (RF; (Breiman, 2001a), both using sklearn v.1.2.2, a linear mixed-effect model (multilevel model regression; MLM; (Goldstein, 2011) using statsmodels v. 0.13.2, mixed-effects random forest (MERF; (Hajjem et al., 2012), using MERF v.1.0.0 and a recurrent neural network using an a Long Short-Term Memory (LSTM; (Hochreiter and Schmidhuber, 1997) architecture using keras v.2.9.0. For precise model formulations see supplement code. The LR and RF are considered the benchmark methods ignoring the time-dependent structure of the data. Linear regression minimises the residual sum of squares in the equation y=Xβ+ϵ where X is the design matrix containing the predictors and y is the symptom outcome. The MLM, MERF, and LSTM all take the time-dependency of the data into account but differ in their estimations of the time effect. MLMs account for the time-dependency as a random effect estimating the within-subject variation over time. In this case, the design matrix familiar from the LR case is extended to the random effects (Z), y=Xβ+Zu+ϵ, fitted using restricted maximum likelihood and the MLM was also optimized for possible quadratic and cubic linear trends. MERFs, on the other hand, leverage their tree-based structure to implicitly capture the interaction effects between time and the variables and estimate the random effects across subjects based on the residual of the predictions. LSTMs were designed for time series and are capable of learning and retaining information over the fullextent of a sequence. While not included in the main comparisons a dummy regressor was also used to establish a minimum prediction score. This dummy regressor assumed only the mean of the outcome in the training sample for each timepoint and was implemented in sklearn v.1.2.2.

#### 2.1.3 Prediction timepoint

**Fig. 2:**
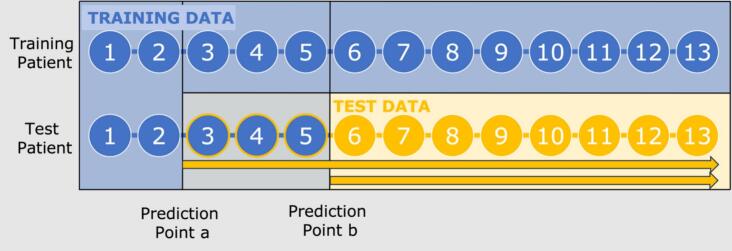
– Model training data Prediction point a) uses the first two timepoints to predict the remining 11 timepoints (short training sequence). Prediction point b) uses the first 5 timepoints to predict the remaining 9 timepoints (long training sequence).

To investigate the benefit of gathering longer sequences, the 13 timepoints were split in two different ways: Firstly, (a) using the initial two timepoints to represent the shortest possible sequence to predict the remaining 11 timepoints (short training sequence). The two timepoints included are the screening and pre-treatment questionnaire, which means this prediction is done before the patient gets access to the treatment content. Secondly, (b) using the pre-treatment timepoints and 3 timepoints during initial treatment (long training sequence), to predict the remaining 9 timepoints, as can be seen in Fig. 2. The latter was determined as a trade-off between keeping as many timepoints as possible and having a prediction early enough in treatment for it to be useful e.g. (Forsell et al., 2019). The prediction was not autoregressive, meaning that the entire remaining sequence was predicted simultaneously.

MLM and MERF approaches had a time-dependent train-test split to estimate the random effects, meaning that the training dataset for each split also contained the test-patients' first two to five timepoints as seen in Fig. 2. This uses the possibility to retrain the less computationally expensive models at the time of prediction to estimate the random effects. The evaluation of these algorithms was not affected as these timepoints were not included. The remaining algorithms did not utilize the initial timepoints for the test patients during training.

#### 2.1.4 Imputation

Imputation of missing data was done before cross-validation in a trade-off between computational resources and expected impact as proposed by (Jaeger et al., 2020). Imputation was carried out in accordance with a multilevel imputation (Grund et al., 2018) with three imputations using MICE implemented in R (Buuren and Groothuis-Oudshoorn, 2011). Each of the three treatments was imputed separately to avoid adding an additional hierarchical level at this step. Three imputation datasets were selected due to subsequent exponentially growing computational requirements. The questionnaires items were imputed (and subsequently summed) due to the superiority of using item imputation compared to scale level imputation (Gottschall et al., 2012). This was achieved with a linear mixed model with predictive mean matching (2 l.pmm) (Van Buuren, 2018). To combine estimations from the different imputations Rubin's rules were used (Van Buuren, 2018), this included the modified standard errors and degrees of freedom of the mean prediction across imputation sets to correct for the variance due to the imputation. Comparisons between models were done based on these means and standard errors.

#### 2.1.5 Validation

We used cross-validation (CV) instead of a single holdout dataset based on results in (Poldrack et al., 2020) as well as our previous research (Hentati Isacsson et al., in press). A nested CV procedure in conjunction with multiple imputation suggests the validity of confidence intervals (Bates et al., 2021). All hyperparameters were tuned in the inner cross-validation loop, to prevent overfitting (Bates et al., 2021; Cawley and Talbot, 2017). The outer CV-loop consists of ten splits and the inner of five. Each of the three imputed datasets - each with a split for the longer and shorter training sequence - went through the 10 × 5 CV loops. The inner CV loop determined the hyperparameter tuning, while the outer CV loops were used to compare the model performances, see Fig. 1.

Due to the computational requirements a limited set of hyperparameters was investigated. LR did not have any hyperparameters. The MLM hyperparameter was whether it was fitted with a linear, quadratic, or cubic time feature. For the RF model, which will be used as a benchmark, the parameters from a previous study with a near identical dataset were used, which had a minimum sample split of 10, and a minimum leaf size of 5 (Hentati Isacsson et al., in press). The MERF model searched for the best parameters of minimum sample split size in [5,10,40] and a minimum leaf size of [2,5,20]. The LSTM model also tested a small setup of parameters due to the computational requirements and the simplicity of the task at hand: the number of layers was set to either one or two, and dropout was set to either 0 or 0.2. For more details see the supplement code. In the internal CV loop, the time-dependent methods were optimized for their Root Mean Squared Error (RMSE) score based on the prediction timepoints (ergo the label width), for example, the first two timepoints were scored based on timepoint 3–13 and using data up to and including timepoint five were scored based on timepoint 6–13.

#### 2.1.6 Prediction measures

While models were optimized for their RMSE score, several other measures were calculated as well, see details in the supplement. Based on the scaling of the symptoms 0–1 the RMSE can be interpreted as the mean percentage wrong in the prediction. An RMSE of 0.1 would equal on average 10 % wrong in predicting the continuous outcome score. Balanced Accuracy (BACC) was also chosen to enable comparison to other models in the field and simplify interpretation, with a BACC of 50 % representing a model not superior to guessing the majority category each time. Thus, a dummy classifier only guessing the majority category would have a BACC of 50 %.

## 3 Results

**Fig. 3:**
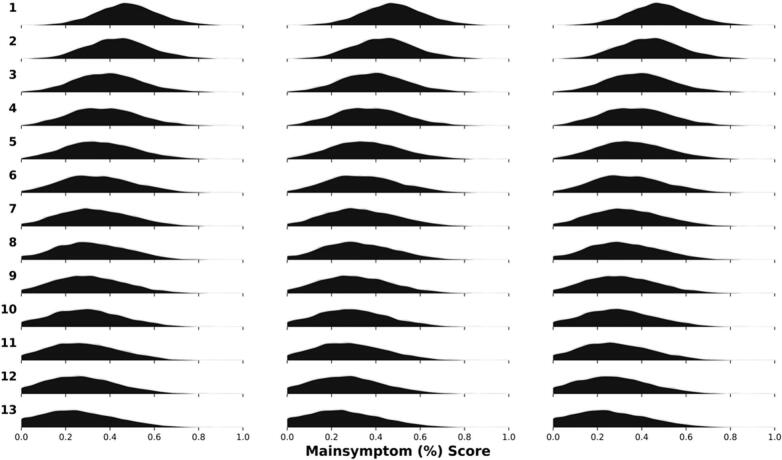
- Symptom distribution over time and imputation sets Density of symptom data across timepoints and the 3 imputed datasets. Very slim differences across the imputed datasets.

See Table 1 patient characteristics, and (Hentati Isacsson et al., in press) for further details. The changes in symptom distributions over time are shown in Fig. 3.

**Table 1:** Baseline characteristics.

Characteristics	Sex	Age (mean (SD))	Education (count (%))^a^	Main Symptom (PRE)^b^ (mean (SD))	Main Symptom (POST)^b^ (mean (SD))
	Female: 4027 (63 %) Male: 2409 (37 %)	35 (11)	Primary: 425 (7 %) Secondary: 2842 (48 %) Postsecondary: 2712 (45 %)	44 % (15 %)	27 % (18 %)

a) *n* = 463 had missing on education b) Main symptom are the primary symptom measure for each treatment before treatment start (timepoint 2) and after treatment completion (timepoint 13) scaled as a percent of maximum possible score. Panic Disorder Symptom Scale-Self Report, Montgomery-Åsberg Depression Rating Scale Self-report, and Leibowitz Social Anxiety Scale-Self report, respectively. Min was 0 for all three questionnaires and max was: 28, 54, 144 for PDSS-SR, MADRS-S, LSAS-SR respectively. Thus 44 % would be in raw points: 12.32, 23.76 and 63.36 for each scale respectively.

**Fig. 4:**
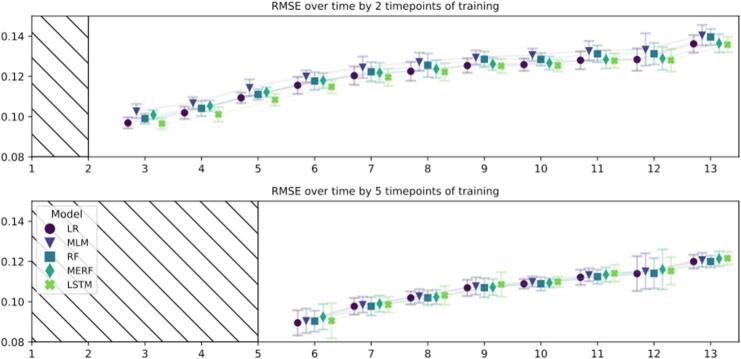
– Prediction Root Mean Squared Error for predicting symptoms during remaining time in treatment. The root mean squared error (RMSE) mean and 95 % CI based on the 3 imputed datasets. The score for each timepoint is the RMSE for predicting that timepoint. The final post-treatment is timepoint 13. The upper panel shows prediction for all progressive weeks based on 2 timepoints of training, the lower panel shows prediction with 5 timepoints of training. LR, Linear Regression. RF, Random Forest. MLM, Multilevel Model regression. MERF, Mixed Effects Random Forest. LSTM, Long Short-Term Memory. MLM, MERF, and LSTM are the time-dependent models. The RMSE can be interpreted as the mean percentage wrong in the prediction.

**Fig. 5:**
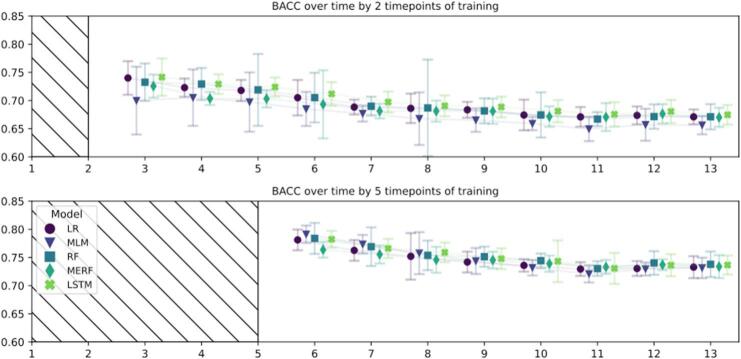
– Prediction Balanced Accuracy for predicting symptoms during remaining time in treatment. The balanced accuracy (BACC) mean and 95 % CI based on the 3 imputed datasets. The score for each timepoint is the BACC for predicting that timepoint. The final post-treatment is timepoint 13. The upper panel shows prediction for all progressive weeks based on 2 timepoints of training, the lower panel shows with 5 timepoints of training. LR, Linear Regression. RF, Random Forest. MLM, Multilevel Model regression. MERF, Mixed Effects Random Forest. LSTM, Long Short-Term Memory. MLM, MERF, and LSTM are the time-dependent models.

The amount of missing over time is detailed in supplement where a link to the full results datafile can be found. Fig. 4, Fig. 5 show the mean and 95 % confidence intervals for RMSE and BACC respectively, for each of the models and timepoints divided by the length of the training sequence. For both training sequences (a) short with two and b) long with five timepoints), prediction results get worse as the weeks are progressively further away from the prediction timepoint, see Fig. 4, Fig. 5.

Predictions for the next week with the short training sequence result in the RSME (mean [SD]) of 0.0992 [0.0026], whereas the post-treatment timepoint in week 12 is 0.1377 [0.022]. For the long training sequence, results show a similar development at 0.0907 [0.0011] at week 5 and 0.1208 [0.0008] at post-treatment.

For the short training sequence, the dummy regressor average RMSE across all timepoints was 0.1680 with negligible variation. In terms of models, the maximum average RSME across all timepoints was at 0.1239 (MLM) and the minimum, hence best, at 0.1186 (LSTM). In terms of BACC, this translates to a range of 67.45–69.95 %. Across all timepoints, the MLM performed slightly worse than the other models; however, on average, by only 0.0053 RSME or 2.5 %-points BACC to the winning model. LSTM performed best across all timepoints, with a negligible distance to the next runner-up (LR), at merely on average 0.0005 RSME or 0.55 %-points BACC.

For the long training sequence, the dummy regressor average RMSE across all timepoints was 0.1694 with negligible variation. In terms of models, the maximum average RSMEs were at 0.1077 (LSTM and MERF), and the minimum, hence best performing, was at 0.1064 (LR). In BACC, this results in a range of 74.42–75.14 %. In the longer training sequence, the dichotomization necessary for calculating BACC changed the order of the models from (worst to best) LSTM, MERF, MLM, RF, LR to MERF, MLM, LR, LSTM, RF. In either setting, the difference between the two worst (0) and two best (0.0002) RSMEs and (0.33) and (0.11) BACC %-points remain negligible.

In summary, the models showed minor variation within the respective settings, including the time-independent models (LR and RF) versus the time-dependent models (LSTM, MERF, MLM). Across all cases, at least one time-independent model performed on par with one of the time-dependent ones. However, all tested models were decidedly better than the dummy regressor.

**Fig. 6:**
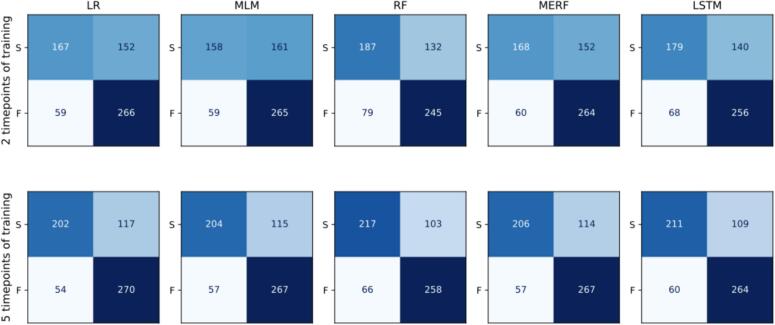
– Confusion matrices for predicting post-treatment outcome based on 2 or 5 timepoints of training. In each matrix: the true positives (upper left), false negatives (upper right), false positives (lower left), and true negatives (lower right) mean based on the 3 imputed datasets. This mean is aggregated by rubins rule can result in minor differences for the total amount of patients in the predicted class across models due to rounding. The first row shows confusion matrices for predicting final outcome based on 2 timepoints of training (short training sequence), the second row shows confusion matrices for predicting final outcome based on 5 timepoints of training (longer training sequence). Each column designated one model: LR, Linear Regression. RF, Random Forest. MLM, Multilevel Model regression. MERF, Mixed Effects Random Forest. LSTM, Long Short-Term Memory. MLM, MERF, and LSTM are the time-dependent models. S, ‘success’ class favourable outcome for the patient, F, ‘non success’ class, not favourable according to our dichtomization criteria.

The longer training sequence (five timepoints) had an average RMSE (mean [SD]) of 0.1071 [0.0006] across models and time, −0.0197 [−0.0223, −0.0172] lower mean [95 % CI] than the shorter training sequence (two timepoints) that had an RMSE of 0.1208 [0.0021]. In terms of BACC, this translates to 68.97 % [0.96 %] for the short training sequence and with a (mean [95 % CI] 6.92 % [5.88 %, 7.96 %] higher mean for the longer sequence with 74.78 % [0.30 %]. See supplement result file for the summarized results. At post-treatment, hence the furthermost point to predict, for the long training sequence, the mean BACC across all models is 73.46 % with models ranging very closely between a minimum of 73.15 % (MLM) and a maximum of 73.79 % (RF). The models perform less well with the short training sequence, with the shorter having a (mean [95 % CI]) -6.58 % [−7.45 %, −5.72 %] smaller mean, but still achieve a BACC of [95 % CI] 65.63 % [64.00, 67.3 %] (MLM), 67.01 % [65.32 %, 68.70 %] (MERF), 67.11 % [65.80 %, 68.42 %] (LR), 67.12 % [64.88 %, 69.37 %] (RF), and 67.46 % [65.73 %, 69.20 %] (LSTM). For a detailed look at the dichotomized prediction results for the post-treatment timepoint see the confusion matrices for all models in Fig. 6.

The improvement in BACC for the longer training sequence compared to the shorter sequence is mainly a decrease in false negatives. The best model for the shorter training sequence (LSTM) achieves a sensitivity of 56.01 % [53.89 %, 58.13 %] and a specificity of 78.91 % [76.65 %, 81.18 %] at post-treatment. For the longer training sequence, the LR has a similar tendency at 63.28 % [60.53 %, 66.03 %] sensitivity and 83.29 % [81.59 %, 84.50 %] specificity, whereas the RF achieves a more balanced result of 67.89 % [65.39 %, 70.39 %] to 79.70 % [74.91 %, 84.48 %]. See supplementary Fig. 2 for sensitivity and specificity graphs.

## 4 Discussion

Our results do not support the claim that time-dependent models improve symptom prediction within the context of ICBT. In the dataset at hand, time-dependent models did not yield significantly better symptom outcome predictions than time-independent models and overall, no superior method could be identified. In fact, the simplest model (Linear Regression; LR) performed almost on par with the most complex one (Long Short-Term Memory; LSTM). The results from (Afshartous and de Leeuw, 2005) showed that the difference between linear regression and a multilevel model decreased as the number of groups and timepoints increased (up to a maximum of 30,000 datapoints). With almost three times more datapoints in our study, this could explain the convergence in results between LR and MLM. This also concurs with findings in (Hajjem et al., 2012) which found a lesser discrepancy between using MERF in comparison to LR, MLM, and RF with predicting points for new groups.

Hence, it is plausible that the number of groups (patients) together with the limited number of datapoints per individual (13) contribute to the initially counter-intuitive results. Specifically, we argue for the possibility that our large number of patients makes the random effect less influential in the predictions, in return causing predictions to converge. With a very small amount of datapoints (two or five) to estimate the random effects for, the predictions are similar to predicting data for unknown groups, thus contributing to the small influence of the random effects. On the performance of LSTM, a study by (Hunt et al., 2017) with up to 240 timepoints and over 34,148 intensive care unit cases found that LSTM performed distinctly better than logistic regression. While not the only factor, this indicates that dataset size could favor LSTM in relation to other models. In a dataset similar to ours a greatly increased number of measurement points would possibly be beneficial. Thus, while many other factors influence model performance, our results hint that our dataset size could influence the comparative performance of the models. As such, our results are part of a line of studies that fail to find more sophisticated and theoretically promising models and features to be superior to simple approaches in similarly sized ICBT datasets (Gogoulou et al., 2021; Hentati Isacsson et al., in press; Zantvoort et al., 2023). This does not bar the possibility of more sophisticated models improving performance, further research should investigate how to optimize models in relation to ICBT data. Specifically, a dataset with more timepoints that are equally spaced in time could improve results. Albeit these findings show that a simpler model that does not account for time dependency can also be a viable alternative if properly validated. Furthermore, while the focus of this study is prediction and not inference (Breiman, 2001b), ignoring nested data structures when using models for inference leads to biased estimates (Afshartous and de Leeuw, 2005; Magnusson et al., 2018). The differences in our findings in relation to inferential models further underscore the delimitation between estimation for inferences and developing models for prediction (Breiman, 2001b; Yarkoni and Westfall, 2017).

We found that using the longer training sequence (5 timepoints) improved RMSE by 1.37 %-points (5.81 % BACC-points) compared to using the shorter training sequence (2 timepoints) across methods, but without any differential effect of time-dependence. This is in line with previous research providing evidence for the predictive value of using longer sequences (Becker et al., 2020) and additional data (Afshartous and de Leeuw, 2005; Hunt et al., 2017; Yang et al., 2017).

Later symptom scores were more likely to be missing than early ones as depicted in Supplementary Fig. 1. The resulting increase in imputed values yields a broader range of predictions across imputed datasets, in turn enlarging the weighted standard error for these timepoints (see Fig. 4). This was most pronounced for timepoint 12. The dichotomization of prediction was largely unaffected by this pattern, albeit having broader standard errors overall, probably due to the imputations falling on the “right side” of the cut-off.

In terms of absolute evaluation, 94 % of all prediction models are above 67 % balanced accuracy, a performance level that has previously been found to be clinically useful (Forsell et al., 2019). However, none of the lower CI-bounds for the shorter training sequence surpassed the preliminary benchmark performance level of 67 % but all lower CI-bounds of the longer training sequence did. At four out of twelve weeks of treatment (day 28), the longer sequence allows sufficient room for a treatment adaptation to shift the treatment trajectory for the patient (Forsell et al., 2019). As a result, we conclude that the minimal data use of only symptom questionnaires can already suffice for predicting treatment outcome, thus serving to make adaptive treatment decisions and subsequently improve outcomes.

These results were validated using repeated multiple imputation and a 10-fold cross-validation with an additional 5 inner folds. Given the dataset size and minimal data, it is plausible to suggest that the predictive capabilities within symptom prediction in ICBT may be around the accuracies and metrics observed in this study. However, the evidence at hand is a mere case study and further research is needed to substantiate the generalizability of the assertions made in this paper. While we explored various hyperparameters during cross-validation, we opted for a simplified LSTM parameter set to accommodate the dataset's limitations. Nonetheless, the possibility of discovering more effective parameters remains. Moreover, employing additional imputed datasets and exploring alternative imputation methods could have enhanced the robustness of our findings. Despite these considerations, we believe our approach strikes a reasonable balance between computational efficiency and methodological complexity. When focusing on symptom data only, the quality of the measures is of great relevance. Considering this relevance, we believe that the measurement and evaluation of symptoms using standardized questionnaires require further development and subsequent investigation regarding their predictiveness (Pendrill, 2018).

## 5 Conclusion

Firstly, our results do not support that utilizing time-dependent models improves symptom outcome predictions compared to time-independent models in the context of Internet-Based Cognitive Behavioral Therapy (ICBT), at least with our dataset size. The results show no clear advantages for complex models such as LSTM. Further, our findings show a convergence in predictive performance between models utilizing a random effect and those that do not. Secondly, using longer training sequences demonstrated a significant improvement in RMSE. This is consistent with previous research emphasizing the value of additional training data. Thirdly, all prediction results using a longer but none of the shorter training sequence significantly surpassed a preliminary benchmark for clinical usefulness of 67 % balanced accuracy. Our results show that accessible and easily implemented models can be used to identify the patients most at risk of not benefitting enough from treatment over the course of treatment.

## Funding

This work was mainly supported by The 10.13039/501100004359Swedish Research Council (VR), The Erling Persson family foundation (EP-Stiftelsen), and The Swedish ALF-agreement between the Swedish government and the county councils, with additional funding by the 10.13039/501100001729Swedish Foundation for Strategic Research (SSF). The funding sources were not involved in any part of the study.

## Declaration of competing interest

The authors declare that they have no known competing financial interests or personal relationships that could have appeared to influence the work reported in this paper.

## Appendix A Supplementary data

Supplementary material Image 1
