# Supplementary material for: Making the most out of timeseries symptom data: A machine learning study on symptom predictions of internet-based CBT
Source: Internet Interv. 2024 Sep 12;38:100773. doi: 10.1016/j.invent.2024.100773 (PMC11416613; doi:10.1016/j.invent.2024.100773)
Supplement: Supplementary file 1 — Supplementary material [file mmc1.docx]

Supplement document:

**Manuscript**

Making the Most Out of Timeseries Symptom Data: A Machine Learning Study on Symptom Predictions of Internet-based CBT

**Note**

This supplement contains more details for the main paper, including link to raw data, and additional figures and results.

The entire code for prediction, model tuning, and result processing can be found at: https://osf.io/bwvg9/?view_only=1248c2d8a8e146e1a5a20f5e59c8cb46

**Contents**

**Missing data**

Supplementary Figure 1

**Sensitivity and specificity**

Supplementary Figure 2

**Supplementary files**

The following files found at: https://osf.io/bwvg9/?view_only=1248c2d8a8e146e1a5a20f5e59c8cb46

1. Code (rar file)

2. Result summary (csv file)

3. Results all metrics (csv file)

4. Codebook csv files. (rar file)

Missing data

| Figure 1 – Missing data |
| --- |
| 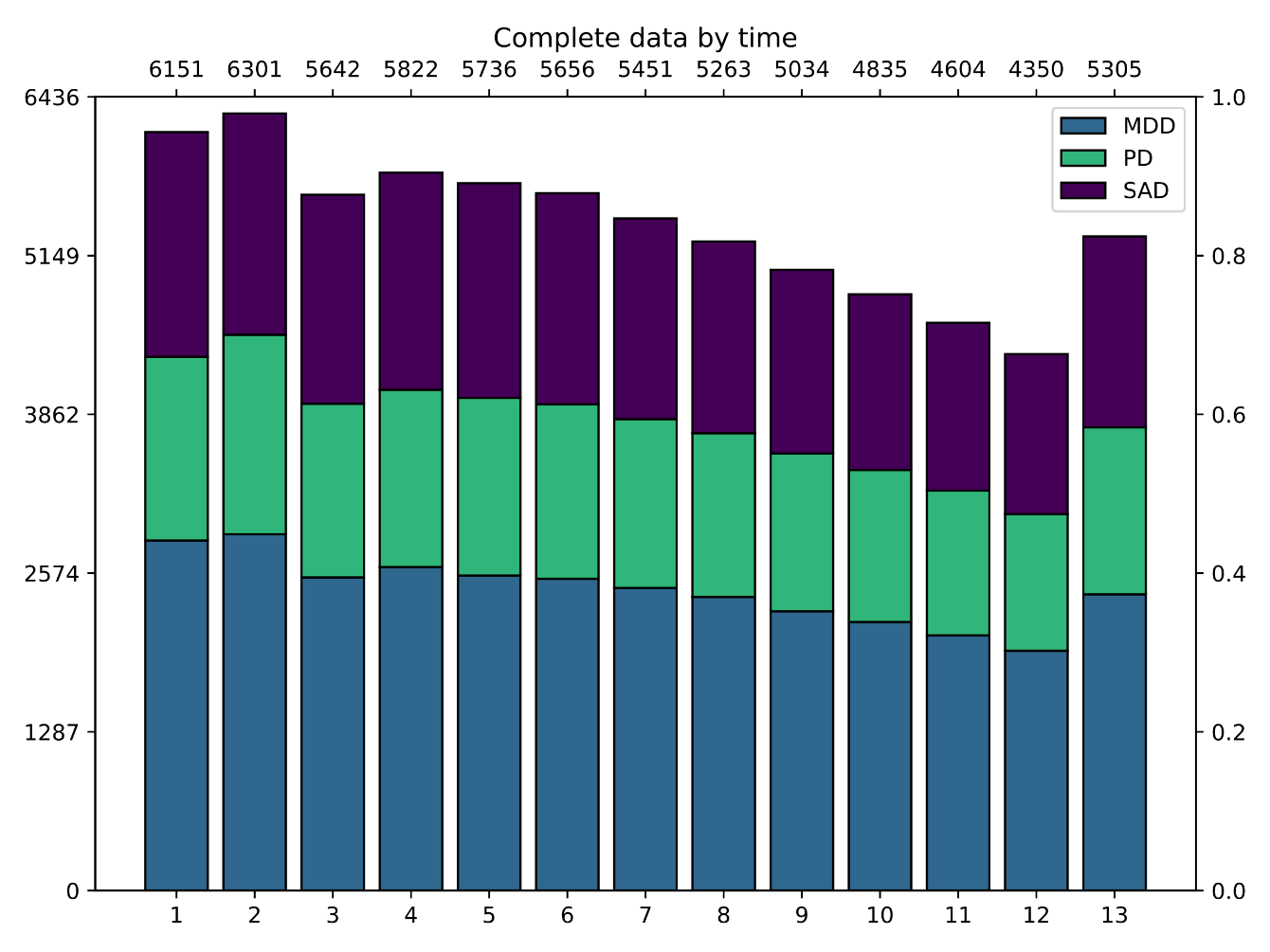 |
| Complete data over the time points. MDD, Major Depressive Disorder. PD, Panic Disorder. SAD, Social Anxiety Disorder. Y- axis to the left shows absolute number of complete cases, y-axis to the right shows fraction. X-axis above shows absolute numbers, and x-axis below show timepoints. Note that missing data was subsequently imputed as detailed in the manuscript. |

Sensitivity and specificity

| Figure 2 – Sensitivity and specificity |
| --- |
| 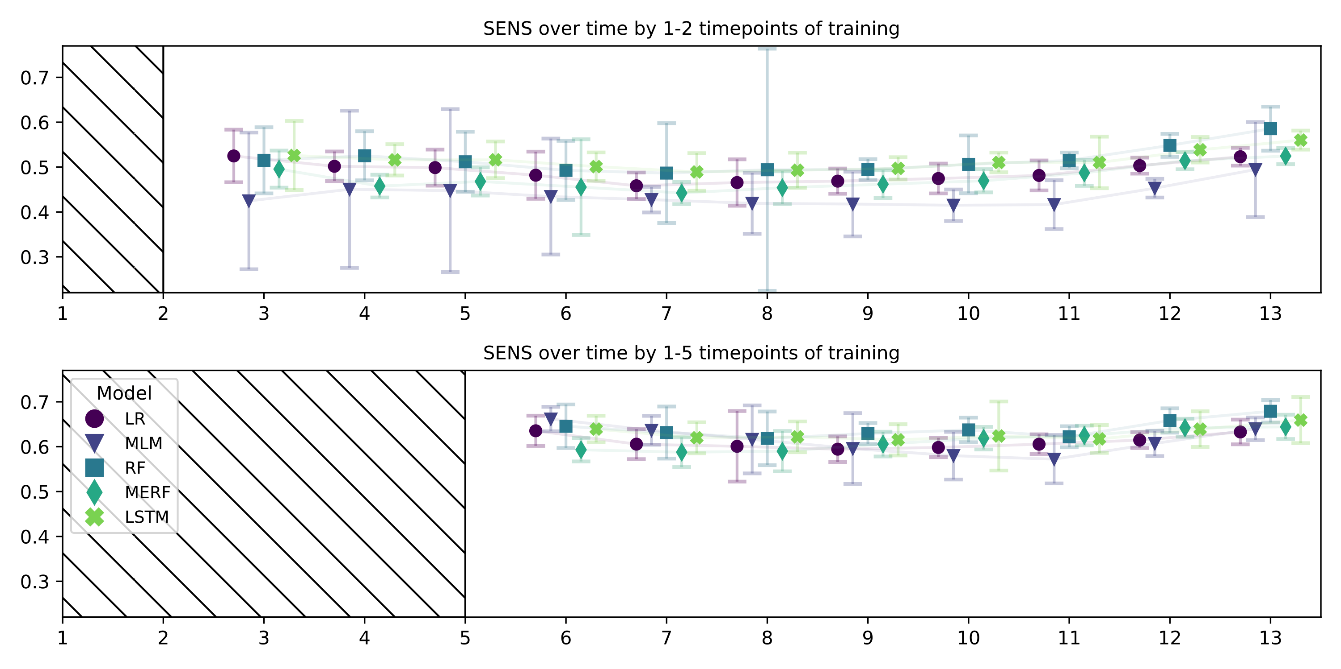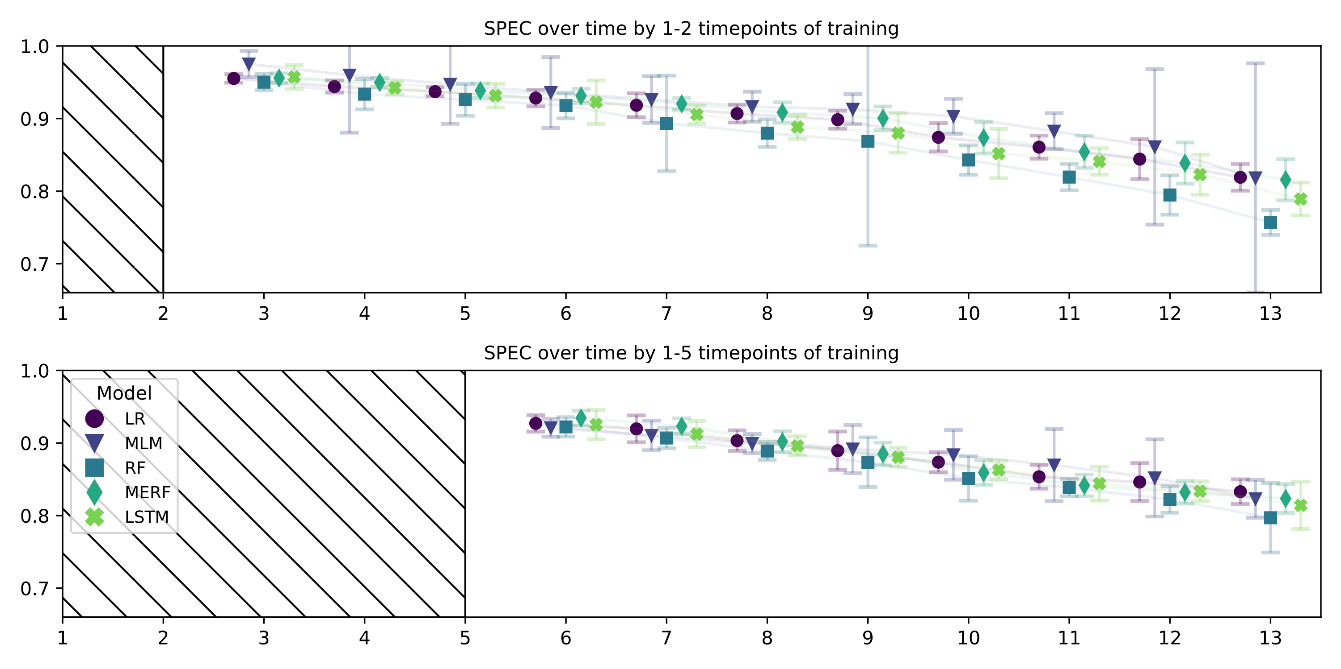 |
| Sensitivity (SENS) and Specificity (SPEC) mean and 95% CI based on the 3 imputed datasets. The score for each timepoint is the SENS/SPEC for predicting that timepoint. The final post-treatment is timepoint 13. The upper panel shows prediction for all progressive weeks based on 2 timepoints of training, the lower panel shows with 5 timepoints of training. LR, Linear Regression. RF, Random Forest. MLM, Multilevel Model regression. MERF, Mixed Effects Random Forest. LSTM, Long Short-Term Memory. MLM, MERF, and LSTM are the time-dependent models. |

References

Fantino, B., & Moore, N. (2009). The Self-Reported Montgomery-Åsberg Depression Rating Scale Is a Useful Evaluative Tool in Major Depressive Disorder. *BMC Psychiatry*, *9*(1), 26. https://doi.org/10.1186/1471-244X-9-26

Furukawa, T. A., Shear, M. K., Barlow, D. H., Gorman, J. M., Woods, S. W., Money, R., Etschel, E., Engel, R. R., & Leucht, S. (2009). Evidence-Based Guidelines for Interpretation of the Panic Disorder Severity Scale. *Depression and Anxiety*, *26*(10), 922–929. https://doi.org/10.1002/da.20532

Glischinski, M. von, Willutzki, U., Stangier, U., Hiller, W., Hoyer, J., Leibing, E., Leichsenring, F., & Hirschfeld, G. (2018). Liebowitz Social Anxiety Scale (LSAS): Optimal cut points for remission and response in a German sample. *Clinical Psychology & Psychotherapy*, *25*(3), 465–473. https://doi.org/10.1002/cpp.2179

Hentati Isacsson, N., Ben Abdesslem, F., Forsell, E., Boman, M., & Kaldo, V. (in press). Methodological choices and clinical usefulness for machine learning predictions of outcome in Internet-based cognitive behavioral therapy. *Communications Medicine*

Karin, E., Dear, B. F., Heller, G. Z., Gandy, M., & Titov, N. (2018). Measurement of Symptom Change Following Web-Based Psychotherapy: Statistical Characteristics and Analytical Methods for Measuring and Interpreting Change. *JMIR Mental Health*, *5*(3), e10200. https://doi.org/10.2196/10200
